# Effects of Alcohol Hangover on Cognitive Performance: Findings from a Field/Internet Mixed Methodology Study

**DOI:** 10.3390/jcm8040440

**Published:** 2019-03-30

**Authors:** Andrew Scholey, Sarah Benson, Jordy Kaufman, Chantal Terpstra, Elizabeth Ayre, Joris C. Verster, Cory Allen, Grant J. Devilly

**Affiliations:** 1 Centre for Human Psychopharmacology, Swinburne University, Melbourne, VIC 3122, Australia; sarahbenson@swin.edu.au (S.B.); cterpstra@swin.edu.au (C.T.); eayre@swin.edu.au (E.A.); 2 Swinburne BabyLab, Swinburne University, Melbourne, VIC 3122, Australia; jkaufman@swin.edu.au; 3 Division of Pharmacology, Utrecht University, 3584 CG Utrecht, The Netherlands; j.c.verster@uu.nl; 4 Queensland Police Service Academy, GPO Box 1110, Archerfield, QLD 4108, Australia; allencorey1966@hotmail.com; 5 School of Applied Psychology and Griffith Criminology Institute, Griffith University (Mt Gravatt Campus), Mt Gravatt, QLD 4122, Australia; grant@devilly.org

**Keywords:** hangover, alcohol, internet, attention, executive function, working memory

## Abstract

Results from studies into the cognitive effects of alcohol hangover have been mixed. They also present methodological challenges, often relying on self-reports of alcohol consumption leading to hangover. The current study measured Breath Alcohol Concentration (BAC, which was obtained via breathalyzer) and self-reported drinking behavior during a night out. These were then related to hangover severity and cognitive function, measured over the internet in the same subjects, the following morning. Volunteers were breathalyzed and interviewed as they left the central entertainment district of an Australian state capital. They were provided with a unique identifier and, the following morning, logged on to a website. They completed a number of measures including an online version of the Alcohol Hangover Severity Scale (AHSS), questions regarding number and type of drinks consumed the previous night, and the eTMT-B-a validated, online analogue of the Trail Making Test B (TMT-B) of executive function and working memory. Hangover severity was significantly correlated with one measure only, namely the previous night’s Breath Alcohol Concentration (*r* = 0.228, *p* = 0.019). Completion time on the eTMT-B was significantly correlated with hangover severity (*r* = 0.245, *p* = 0.012), previous night’s BAC (*r* = 0.197, *p* = 0.041), and time spent dinking (*r* = 0.376, *p* < 0.001). These findings confirm that alcohol hangover negatively affects cognitive functioning and that poorer working memory and executive performance correlate with hangover severity. The results also support the utility and certain advantages of using online measures in hangover research.

## 1. Introduction

The alcohol hangover (AH) is defined as “the combination of mental and physical symptoms, experienced the day after a single episode of heavy drinking, starting when blood alcohol concentration approaches zero” [1]. It describes a feeling of malaise that follows a bout of drinking when blood alcohol levels are at or returning to zero [2,3,4,5,6,7]. The AH is variously characterized by somatic and behavioral symptoms including headache, thirst, stomach upsets, negative mood, and cognitive problems.

There can be considerable inter-individual variability in the pattern, severity, and temporal characteristics of hangover symptoms [8], with no clear relationship between AH severity and any single physiological process (although cytokine response to alcohol is emerging as a possible key factor [9,10]). Other mechanisms that may contribute to AH include, but are not limited to, gut dysbiosis (including ghrelin-mediated), decreased blood glucose concentrations, poor sleep architecture, dehydration (and concomitant electrolyte imbalances), oxidative stress, and inflammatory responses [2,11,12]. These last two may in part be elevated in response to circulating ethanol metabolites.

The majority of previous research exploring the cognitive effects of alcohol has focused on acute intoxication, and the long-term neurocognitive consequences of alcohol dependence [7,13,14]. Acute intoxication impairs aspects of memory, attention, and psychomotor performance [15,16,17,18,19,20]. Alcohol also produces a characteristic shift in the speed/accuracy trade-off. Unlike other impairing drugs which tend to slow responding, alcohol typically increases error rates with little effect on response speed [16,17,21].

Compared with alcohol intoxication, relatively little research has been directed at the specific cognitive effects of AH [22,23]. A recent meta-analysis of next-day cognitive effects of heavy alcohol consumption included 19 studies published in 11 articles since 1970 [24]. It concluded that sustained attention, short- and long-term memory, and psychomotor speed are the cognitive domains most susceptible to hangover.

Any hangover-related cognitive impairment could have major implications for everyday activities. For example, hangover impairments to driving ability were similar to those observed at Breath Alcohol Concentrations (BAC) of 0.05–0.08% [25], that is similar to those. Such impairments have clear ramifications for safety-sensitive occupations, but also negatively impact on those that continue to engage in everyday activities while in a hangover state. In the context of absenteeism and presenteeism, it has been estimated that alcohol hangover costs the US economy 179 billion annually in lost productivity [26].

There are a number of methodological approaches to the study of hangover effects on cognition (see Stephens et al., 2014 for a critical review) [2]. These include laboratory studies where controlled doses of alcohol are administered, usually in a relatively pure form (typically vodka), and cognitive outcomes are measured once BACs have returned to zero. Alcohol is either administered at fixed doses or titrated to reach a target BAC. This approach has the advantage of providing relatively high levels of control, particularly regarding the timing of alcohol administration and measurement of physiological and functional endpoints. On the other hand, it may not have high ecological validity. For example, in real-life drinking situations individuals may consume a variety of beverages over different lengths of time. Secondly, because Ethics Boards tend to err on the side of caution, laboratory studies typically use lower levels of alcohol than those observed in the field. Even when bar-like settings are simulated in the laboratory there tends to be a limit on target alcohol levels.

An alternative methodology in AH research is to use a so-called ‘naturalistic’ design. Here participants visit the laboratory on two mornings, one after a night’s drinking and another after a sober night (with order counterbalanced across participants) [27]. This method has the advantage of ecological validity by not limiting participants’ drinking. On the other hand, the approach relies on recollection of levels of alcohol consumed to generate an estimated Blood Alcohol Level (eBAC). Given that alcohol intoxication is associated with memory problems (as may be hangover), this is problematic when trying to explore the relationship between alcohol consumed and functional consequences of AH.

The relative utility of these approaches is illustrated by disparate findings regarding cognitive impairments associated with hangover [28]. For example, psychomotor deficits associated with AH were observed in naturalistic studies [23,29,30] but not in laboratory settings [31,32,33].

The current study took a somewhat different approach. Over the past decade or so, internet studies have been increasingly used in psychological research, including to evaluate the impact of substance use and complement field and laboratory studies on the effects of recreational drugs and alcohol [14,16,17,34,35,36,37,38,39,40,41,42]. Here, we employed a mixed methodology involving a subgroup of individuals who were taking part in ongoing field studies, SmartStart [43] and Last Drinks [44], into patterns and consequences of alcohol consumption. These studies included a cohort of patrons who were breathalyzed while leaving the entertainment area of an Australian capital city and agreed to be contacted the following morning to complete an internet study of hangover. This included a version of the Alcohol Hangover Severity Scale (AHSS [28]) and an online analogue of the Trail Making Test B (TMT-B), named the eTMT-B-a test of psychomotor function, working memory, and executive function. This task was chosen as it is relatively brief, the task demands are easily understood, and it captures elements of the major cognitive domains (psychomotor function, attention, and executive function) affected by AH [24,45].

This approach has several advantages. Next-day symptomatology and performance can be linked to measured alcohol levels and drinking characteristics, collecting data via the internet is relatively convenient in that it does not require travelling to a testing location, and the method can address certain methodological questions—for example what factors contribute to attrition in hangover studies.

We hypothesized that previous night BAC would be related to both worse hangover severity and cognitive performance as measured using the eTMT-B. Further analyses explored the influence of beverage types and patterns of drinking on hangover severity and cognitive performance.

## 2. Experimental Section

### 2.1. Pilot Study

A pilot study was conducted to evaluate the validity of the online eTMT-B. Twenty-four young adult volunteers (17 female, mean age 30.29 years, SD 5.03) completed both the pencil-and-paper and online versions of the test (with order counterbalanced across participants).

The traditional TMT-B requires participants to draw lines connecting 25 circles distributed on a page containing single digits and letters. Correct completion involves joining the stimuli alternating between ascending numbers and letters (e.g., 1-A-2-B-3-C…, etc.), with completion time as the main outcome [29]. The TMT-B was administered according to the published protocol [46], with the exception that errors were not corrected (as this would not be possible in the online version).

**Figure 1:**
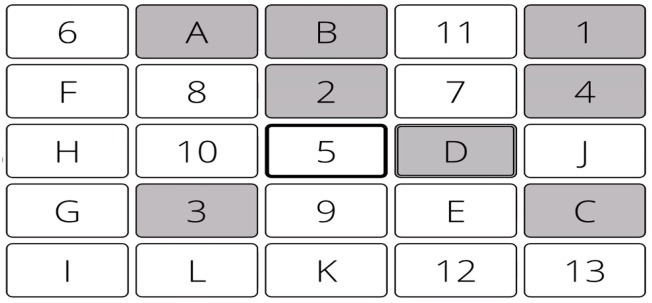
Layout of the online analogue of the Trail Making Test B, eTMT-B. The task requires participants to click ascending numbers and letters alternating between numbers and figures (i.e., 1-A-2-B-3-C, etc.).

The eTMT-B was designed as an online analogue of the Pencil-and-Paper TMT-B. It consisted of a 5 × 5 grid of rectangular panels each labeled with a digit or number. Similar to the paper version, the task involved clicking on alternating ascending numbers and digits starting with 1-A and so on (see Figure 1). Once a panel was pressed it changed color from white to grey, after which clicking on it had no effect. If a participant attempted to press an incorrect button, no response could be made and an incorrect response was recorded. Both versions were scored for errors and completion time.

### 2.2. Main Study

#### 2.2.1. Design

This study formed part of a larger series of studies aimed at determining patterns and drivers for drinking in and around an Australian state capital. This part of the study employed a mixed methodology approach, whereby individuals were approached as they left the Central Entertainment District (CED) and then contacted the following morning to conduct an internet study.

The study was approved by the ethics committees of both Griffith University (2015/704) and Swinburne University (2016/167).

When first engaged during the CED phase, participants were presented verbally with a summary of the nature of the study and what their involvement would entail. Survey and breathalyzer measures were completed only if the participant provided verbal consent for both the concurrent and next morning measures (as approved by the ethics committee) as is usual for this kind of research. Each participant was given a unique identifier card with a link to the study information sheet (www.last-drinks.com.au). They were informed that they would be allowed to withdraw their data (using the anonymous ID number given on the card) and obtain further information regarding the study if they so wished.

#### 2.2.2. Participants

One hundred and five participants provided usable datasets. They were recruited at and around exit points (taxi ranks and train station) in the central entertainment area of Brisbane, Queensland, Australia.

#### 2.2.3. Breath Alcohol

Breath alcohol levels were measured using an Alcolizer LE5 (Alcolizer Pty Ltd, Australia). This device is used by law enforcement agencies throughout Australia and South East Asia, is Australia Standard 3547 certified, and has an accuracy of greater than 0.005 at 0.100 BAC g/100 mL. It has been demonstrated to have high reliability and validity for measuring intoxication in a sample of people attending nighttime entertainment districts [47].

#### 2.2.4. Online Measures

A website survey was constructed which included questions collecting demographic and morphometric information (birth year, gender, weight, and height). Participants were asked specifically about the number of beers/ciders, glasses of wine, shots (unmixed), and alcohol mixed with either energy drinks or other beverages they had consumed the previous night and on a typical night out. This was followed by an 11-item version of the Alcohol Hangover Severity Scale (AHSS [28]), comprising of 11-point scales with endpoints (0 and 10) labelled as ‘absent’ and ‘extreme’. Individual items were ‘sweating’, ‘confusion’, ‘thirst’, ‘nausea’, ‘fatigue (being tired)’, ‘heart pounding’, ‘dizziness’, ‘shivering’, ‘clumsiness’, ‘apathy (lack of interest/concern)’, and ‘stomach pain’. Note that the ‘difficulty concentrating’ item was omitted to reduce expectancy effects while engaging in a task requiring an element of focused attention.

The next sections asked questions regarding the previous night’s alcohol consumption (specifically numbers and types of beverages), sleep, and qualitative hangover data (these will be reported elsewhere). Alcohol intake data were collected according to the number of standard drinks consumed for each drink type (an Australian standard drink contains 10 grams of alcohol). Participants were provided with a link to the Better Health Channel online drink calculator where they were shown images of glasses containing an Australian standard drink. Participants were able to compute the number of standard drinks they had consumed by moving a slider to add or remove alcohol from the glass. Calculations were based on the average alcohol content for each drink type. For example, red wine was calculated at 13% alcohol, white wine at 11.5% alcohol, and full-strength beer at 4.8% alcohol. The full list of average alcohol contents by drink type can be found at http://mapi.betterhealth.vic.gov.au/saywhen/my-drinking/calculator.

Participants were then directed to a website with the eTMT-B. Following completion of the online test, a debriefing page was displayed, and participants were given details for entry to an online competition to win an iPad. They were also compensated with a $15 AUD iTunes voucher if they provided an email address.

#### 2.2.5. Procedure

Individuals were recruited by being approached as they left the central entertainment area of Brisbane, Queensland. If willing to be interviewed, they provided consent, were breathalyzed, and gave details regarding their consumption of alcohol (including time drinking and number of drinks) that night. Those with BACs around 0.05% or above were asked if they agreed to be contacted the following morning. Those consenting (*N* = 346) provided their contact details and estimated bedtime for that night. Participants were given a card with a unique identifier which allowed anonymous linking of their BAC and drinking data to the data collected over the internet.

The following morning individuals were contacted by text message 8 h following their estimated bedtime and again 6 h later if they had not completed the survey. If participating, they entered their unique identifier and then completed the webpages as described above.

#### 2.2.6. Statistics and Analysis

Number of drinks recorded were converted to standard drinks (one Australian standard drink is equivalent to 10 g alcohol). Initial analyses involved exploring the relationship between BAC, hangover severity, and cognitive performance with various drinking factors. These included number of standard drinks, time drinking, and number of specific types of drink. The relationships between these factors were analyzed using Pearson’s correlations. To determine whether BAC affected participation in the hangover part of the study, *t*-tests compared BACs of those who did and did not participate in the next-day online phase of the study.

## 3. Results

### 3.1. Pilot Study

Response times were similar between the paper-and pencil and internet platforms. With a mean completion time of 40.27 s (range 14.71–71.59) for the paper version of the TMT-B and 41.49 s (23.02–75.10) for the eTMT-B (t(23) = 0.116, *p* = 0.909). These were significantly correlated between individuals completing the two platforms (*r* = 0.499, *p* = 0.013).

Published norms for completion of the paper version of the task are typically around 50 s [44,48]. Considering our sample were predominantly students and the paper version involves error correction, our figures are in line with normative data. Errors were rare on both platforms with 4 people making errors on the paper version and 10 people on the eTMT-B. The vast majority of these were single errors.

### 3.2. Main Study

Of 346 participants who were breathalyzed and indicated that they may be prepared to participate in the next-day phase of the study, 105 provided complete online datasets. Sample characteristics of this cohort are presented in Table 1.

Drinking characteristics of the cohort are presented in Table 2. The sample had a mean BAC of 0.11% (SD ± 0.40). They reported drinking for an average of 7.45 hours and had consumed a mean number of 13.5 standard drinks. Analyses of next-day reports revealed that the most consumed drink was alcohol mixed with non-energy drink mixers (*N* = 83 drinkers, consuming a mean of 7.27 drinks) followed by beer/cider (*N* = 59, mean = 7.16), shots (*N* = 43, mean = 3.51), wine (*N* = 31, mean = 5.68), with alcohol mixed with energy drinks being the least consumed beverage both in terms of number of drinkers and average number of beverages (*N* = 30, mean = 2.87).

**Figure 2:**
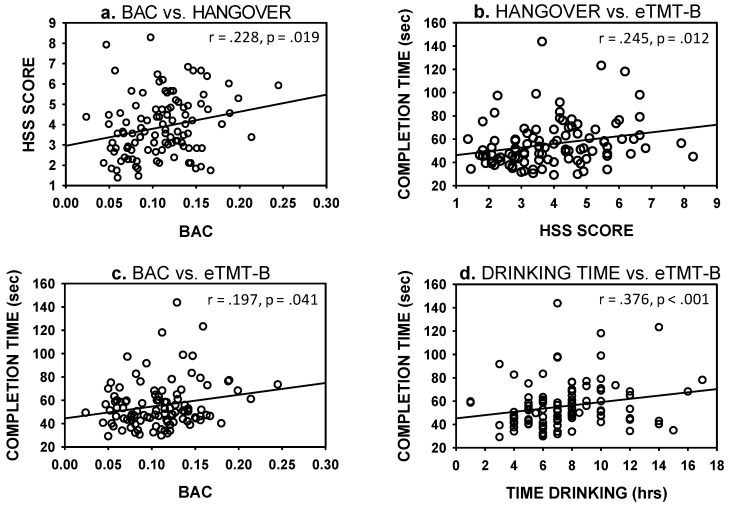
Graphs depicting significant associations between (**a**). previous night’s breath alcohol content (BAC) and hangover severity (Hangover Severity Scale (HSS) score), (**b**). hangover severity and cognitive performance (eTMT-B), (**c**). BAC and cognitive performance (**d**). drinking time and cognitive performance.

The main focus of the study was to examine factors associated with hangover severity and cognitive performance (see Figure 2).

Hangover severity was significantly related to one measure only, namely BAC (*r* = 0.228, *p* = 0.019). The correlation between total number of standard drinks and HSS gave a value of *r* = 0.184, *p* = 0.064. Speed of completion of eTMT-B correlated with self-rated hangover severity (*r* = 0.245, *p* = 0.0120, previous night’s BAC (*r* = 0.197, *p* = 0.04), and drinking time (*r* = 0.376, *p* < 0.001).

Further analyses revealed that BAC correlated significantly with number of standard drinks consumed (*r* = 0.486, *p* < 0.001) and time drinking (*r* = 0.376, *p* < 0.001). Examination of the relationship between individual drinks and alcohol levels revealed significant correlations between BAC and amount of alcohol consumed as beer/cider (*r* = 0.361, *p* = 0.005), wine (*r* = 0.398, *p* = 0.026), and alcohol mixed with other beverages (*r* = 0.228, *p* = 0.038) but not between BAC and shots alone or alcohol mixed with energy drink (Table 3).

Time drinking significantly correlated with standard drinks consumed in total (*r* = 0.633, *p* < 0.001), and as beer/cider (*r* = 0.503, *p* < 0.001) and alcohol mixed with other beverage (AMOB, *r* = 0.257, *p* = 0.019). Standard drinks significantly correlated with drinks consumed as beer/cider (*r* = 0.623, *p* < 0.001), wine (*r* = 0.437, *p* = 0.014), and AMOB (*r* = 0.369, *p* = 0.001). There were no other significant correlations.

There was no difference in the BACs of those who did (*x* = 0.11% ± SD 0.0408) and did not (*x* = 0.11% ± SD 0.0405) complete the online hangover phase of the study (t(345) = 0.240, *p* = 0.81).

## 4. Discussion

Using mixed field and internet methodology, we found that hangover severity is significantly related to BAC and both are associated with worse performance on the eTMT-B test of attention and executive function. Drinking time was also associated with BAC and with worse performance on the task (though not with hangover score).

The current methodology allowed the measurement of BACs and next morning collection of data regarding types of alcohol consumed, evaluation of severity of alcohol hangover, and cognitive functioning. The study confirmed that previous night’s BAC was significantly associated with hangover severity. No other measure was significantly correlated with hangover scores despite some intercorrelations with other measures and BAC including type of beverage consumed.

It has been suggested that different types of alcoholic beverage may influence hangover severity. In particular it has been suggested that non-alcohol constituents of drinks, known as congeners, may differentially affect AH [49]. In particular, it has been suggested that congener-rich drinks such as whiskey produce worse hangover symptoms than beverages with essentially no congeners, such as vodka, although there is little systematic research in this area. Our (albeit limited) data do not suggest that different alcohol types contribute differentially to AH symptomatology. Similarly, different types of mixers had no differential effect on hangover (symptom) severity.

Conversely there was some evidence that certain drink types were more closely associated with BAC. As well as correlating with amount of time drinking, BAC was related to the amount of beer/cider, wine, and AMOBs reportedly consumed (but not shots, or alcohol mixed with energy drinks—AMED). It seems unlikely that the nature of beverage consumed could differentially contribute to BAC. AMOBs were the most commonly consumed drinks (by *N* = 83 people, consuming $\bar{x}$ = 7.27 standard drinks), followed by beer/cider (*N* = 59, $\bar{x}$ = 7.16), and shots (*N* = 43, $\bar{x}$ = 3.51). AMEDs were the least consumed beverages (*N* = 30, $\bar{x}$ = 2.87), and although wine was consumed by a similar number of people (*N* = 31), on average it was consumed at a higher level ($\bar{x}$ = 7.16 drinks). Thus, our data confirm other findings using other methodology [50] showing that irrespective of the type of alcoholic drink or mixer, the most meaningful association was between the number of drinks consumed and BAC.

The error rate on the eTMT-B was generally low, with more than half of the sample (54%) making no errors, and around one quarter (26%) making one or two errors only. This suggests that participants engaged with the eTMT-B and understood task demands of the online version. This confirms the results of the pilot study where there was high correspondence between the paper and online versions of the task. Thus AH-related impairment was largely manifest by slowed function. A pattern of slower reaction times with increasing hangover severity would differentiate alcohol hangover from alcohol intoxication. The latter is typified by a characteristic shift in the speed/accuracy trade-off (SATO) with intoxication leading to more errors while having relatively little effect on reaction times [16,17,21,51]. Since few errors were made on either version of the TMT-B however, we cannot draw strong conclusions from this limited dataset. Nevertheless, the literature does suggest that slowing of response times during AH is more robust than increased errors [24,52]. One focus of this study was to implement a cognitive task that was sensitive to impairment and could be used online (and thus be relatively brief with clear task demands). Clearly, future studies would benefit from a more comprehensive assessment of working memory and executive functions as well as other cognitive domains.

The current study had certain advantages over previous studies applying naturalistic methodology. For example, rather than relying on next-day recall of previous night’s drinks, we had an objective measure of BACs. Additionally, the number of alcoholic beverages consumed and length of time drinking were recorded on the night, making these data less susceptible (though not invulnerable) to recall bias.

While we can be confident that measured BACs were accurate [47], we do not know which phase of drinking participants were in when breathalyzed. Specifically, we do not know if BACs were measured at peak alcohol levels or during the rising or falling limb of the blood alcohol curve. Further, although the BAC measurement occurred at Central Entertainment District exit points (taxi ranks and train stations), we cannot preclude the possibility that some participants continued to consume alcohol, which would affect hangover and related functional consequences.

Another issue is that approximately one third of the sample did not attempt the next-day measures. Drop-out rates in hangover studies are typically high (e.g., 70 % attrition in Grange et al. [53]), suggesting that the current methodology is as viable as others in this respect. One possibility is that those subjects who consume the most alcohol and/or have the worse hangover symptoms are less likely to complete the next-day measures. The current methodology allowed us to address this directly by comparing the BACs of those who did and did not complete the hangover part of the study. This showed that the BACs were more-or-less identical. Furthermore, the sample who completed the next-day measures had a large range of BACs (up to 0.245%), suggesting that reaching a relatively high BAC did not affect the ability to complete the next-day measures. This may be an advantage of the current methodology, as next-day measures were completed online and so were relatively convenient.

The similarity of BACs between the next-day completers and non-completers also partially addresses a potential ethical issue in this type of research, specifically pertaining to consent from intoxicated individuals. It is reasonable to assume that any individual who consented in error would simply not enter into the next-day data collection phase. The fact that previous night’s BACs were statistically similar between those who did and did not complete next-day measures strongly suggests that level of intoxication did not affect consent to the extent that the sample were ‘self-selecting’ in this context.

## 5. Conclusions

In conclusion, using novel methodology, this study has confirmed that higher BACs and associated measures result in worse hangover symptoms and poorer performance on a newly-validated online measure of working memory and executive function—the eTMT-B. Such hangover-related impairments are likely to have clear, real-life ramifications. For example, they would impact on the ability to engage in complex behaviors and may explain some of the hangover-associated impairment of driving [25]. They are also likely to impinge on fundamental aspects of cognitive functioning. Moreover, our findings suggest that a mixed field/internet approach provides a novel and viable methodology for hangover research.

## Figures and Tables

**Table 1:** Demographic and morphometric data of sample. Apart from gender figures are means with range in parentheses.

*N*	105
Males/Females (%)	51.4/48.6
Age	24.7 (17–49) years
Weight	74.0 (43–115) kg
BMI	24.15 (16.58–40.28)

**Table 2:** Reported drinking characteristics, including types of drinks consumed on the night of data collection, Breath Alcohol Concentration (BAC), and hours drinking.

	*N*	Mean	SD	Max
*Drinks consumed*				
Beer/cider	59	7.16	5.86	30
Wine	31	5.68	4.56	20
Shots (unmixed)	43	3.51	3.21	16
Alcohol mixed with Energy Drink	30	2.87	1.98	8
Alcohol mixed with Other Beverage	83	7.27	5.80	40
Total	105	13.48	5.94	35
*Drinking measures*				
BAC (%)	-	0.110	0.040	0.25
Hours drinking	-	7.45	4.09	17

**Table 3:** Correlations between previous night’s BAC, hangover severity, and drinking characteristics.

	BAC (%)	Time Drinking (h)	HSS Score	Standard Drinks (*N*)	Beer/Cider (*N*)	Wine (*N*)	Shots (*N*)	AMED (*N*)	AMOB (*N*)
***N***	105	105	105	102	59	31	43	30	83
**BAC (%)**	-	0.376 ***	0.228 *	0.486 ***	0.361 **	0.398 *	−0.028	−0.099	0.228 *
**Time drinking (h)**	-	-	0.148	0.633 ***	0.503 ***	0.223	0.166	0.068	0.257 *
**HSS score**	-	-	-	0.184	0.171	0.293	−0.010	0.019	0.018
**Standard drinks (*N*)**	-	-	-	-	0.623 ***	0.437 *	0.249	0.360	0.369 **
**Beer/cider (*N*)**	-	-	-	-	-	−0.388	−0.047	0.033	−0.255
**Wine (*N*)**	-	-	-	-	-	-	−0.131	0.162	−0.284
**Shots (*N*)**	-	-	-	-	-	-	-	0.102	−0.067
**AMED (*N*)**	-	-	-	-	-	-	-	-	−0.023

BAC = Blood Alcohol Content, HSS = Hangover Severity Scale, AMED = alcohol mixed with energy drinks, AMOB = alcohol mixed with other beverage. Significant correlations are indicated in bold (*, *p* < 0.05; **, *p* < 0.01; ***, *p* < 0.0001). Drinking characteristics including types of drinks consumed reported on the night of data collection, BAC, and hours drinking.
